# 805 A Simple Technique to Create Partial-Thickness Wounds and Harvest Split-Thickness Skin Grafts in Rat Models

**DOI:** 10.1093/jbcr/irae036.345

**Published:** 2024-04-17

**Authors:** Deborah Choe, Rendell Bernabe, Anahit Simonyan, Kimberly K Gokoffski, Justin Gillenwater

**Affiliations:** Keck School of Medicine of USC, Los Angeles, CA; Virginia Commonwealth University, Richmond, VA; University of Southern California, Los Angeles, CA; University of Southern California, Loa Angeles, CA; Keck Medicine of USC, Los Angeles, CA; Keck School of Medicine of USC, Los Angeles, CA; Virginia Commonwealth University, Richmond, VA; University of Southern California, Los Angeles, CA; University of Southern California, Loa Angeles, CA; Keck Medicine of USC, Los Angeles, CA; Keck School of Medicine of USC, Los Angeles, CA; Virginia Commonwealth University, Richmond, VA; University of Southern California, Los Angeles, CA; University of Southern California, Loa Angeles, CA; Keck Medicine of USC, Los Angeles, CA; Keck School of Medicine of USC, Los Angeles, CA; Virginia Commonwealth University, Richmond, VA; University of Southern California, Los Angeles, CA; University of Southern California, Loa Angeles, CA; Keck Medicine of USC, Los Angeles, CA; Keck School of Medicine of USC, Los Angeles, CA; Virginia Commonwealth University, Richmond, VA; University of Southern California, Los Angeles, CA; University of Southern California, Loa Angeles, CA; Keck Medicine of USC, Los Angeles, CA

## Abstract

**Introduction:**

Developing reliable methods that can generate partial-thickness (PT) wounds of consistent sizes in rats would be a valuable tool for testing and developing novel strategies for wound healing. The process of harvesting a split-thickness skin graft (STSG), which also creates a cutaneous wound, in rats is difficult due to their skin laxity and small size. Current methods in the literature can be time-consuming, require additional supplies, or cannot be performed by a single operator. We propose a novel one-person solution that uses a circular dermatome. We further validate this technique’s reliability in creating STSGs/PT wounds of consistent thickness in rats.

**Methods:**

Technique: Donor site fur was trimmed with electric clippers. The rat was firmly grasped by the non-dominant hand so as to ensure that the donor site skin was taut. Lubricating gel was applied to the donor site. The circular dermatome, set to the desired depth, was held in the dominant hand. Downward, constant pressure was applied with the circular dermatome, with the blade first making contact with the planned donor site's rostral end. Maintaining pressure, the dermatome was pulled caudally until the desired STSG donor site size was reached.

**Validation:** To assess this method's reliability in creating STSGs of consistent thickness, skin grafts (with the device set to 0.001 in.) were harvested from the left bony, left non-bony, right bony, and right non-bony dorsal thoraces on two rats for eight total samples. Samples were fixed in 4% paraformaldehyde, cryosectioned, stained with hematoxylin/eosin, and imaged at 20X. Using an image processing program, 40 thickness measurements (in.) were taken along each sample length. Coefficients of variation (CV) were calculated and compared between samples. CV thresholds for levels of reliability were defined as the following: CV< 10% (very good), CV=10-20% (good), CV=20-30% (fair), and CV>30% (poor).

**Results:**

All four grafts harvested from bony thoraces had CV values between 10-20%. Three of the four grafts harvested from non-bony thoraces had CV values between 10-20% and one had a CV< 10%. Among the grafts with CV values between 10-20%, one graft harvested at a bony site had a CV< 15%, while three grafts harvested at non-bony sites had a CV< 15%. The average time required to harvest one graft was five minutes, with an average of five seconds for active circular dermatome use.

**Conclusions:**

This simple technique can be employed to harvest STSGs and create PT wounds in rat models with overall good thickness consistency. Using this device on non-bony donor sites may be preferred for maximizing intra-graft thickness consistency. This proof-of-concept study requires further validation to optimize STSG donor/PT wound site selection.

**Applicability of Research to Practice:**

This single technique is suitable for both wound healing and STSG studies, allowing researchers to optimize efficiency without compromising reliability.

